# Supplementary material for: Late initiation of antenatal care visit amid implementation of new antenatal care model in Sub-Saharan African countries: A multilevel analysis of multination population survey data
Source: PLoS One. 2025 Jan 31;20(1):e0316671. doi: 10.1371/journal.pone.0316671 (PMC11785278; doi:10.1371/journal.pone.0316671)
Supplement: Attachment — Submitted filename: Response to Reviewers.pdf [file pone.0316671.s003.pdf]

Dear Academic Editor,

Thank you for the opportunity to revise our manuscript. “Late initiation of antenatal care visit amid implementation of new antenatal care model in Sub-Saharan African countries: a multilevel analysis of multination population survey data (ID: PONE-D-24-21779R1)”. We have addressed the concerns raised by the reviewers using a point-by-point response as stated below. The amendments made to the manuscript have been presented using track change in the attachment titled “Revised Manuscript with Track Changes”.

Response to Reviewer #1 Comment

Comment 1: Data source: The authors should provide the accession number or the direct web link in the Availability of Data and Materials section of your manuscript.

Response 1: Thank you very much for your suggestion. We have provided the web link and a reference number of an authorization latter to access the data used in our analysis.

Response to Reviewer #2 Comment

Comment: The authors have addressed all the comments given in the revision one of the manuscript. I have no further comment.

Response: Thank you very much for accepting our manuscript in its current form.

Response to Reviewer #3 Comments:

The paper addresses an important topic. However, it has some major technical weaknesses. In particular, some crucial details on methodology are missing, making it impossible to assess appropriateness of the analysis presented.

Comment 1: It is stated that the analysis is based on multilevel analysis, yet it is unclear what the different levels are. For instance, is the multilevel analysis based on two-levels or 3-level logistic regression, and what are the levels (community, country, etc)? The results presented only show fixed effects without any random effects, so it is unclear whether there were any significant variations across the different levels of analysis. It is impossible to ascertain what multilevel models were fitted, or results based on single-level logistic regression.

Response 1: Thank you very much for your important concern. We have presented the random effect analysis result in the revised manuscript (i.e. we have revised the method and result section based on the suggestion).

Comment 2: It is stated that weighting was undertaken for pooled data, but unclear how this was done. Was weighting based of DHS-derived country-specific weights, or were pooled weights derived, taking into consideration different sampling fractions used by DHS in different countries? The DHS-derived weights are useful for country-specific analysis to achieve national representativeness, but would not be appropriate for pooled data across different countries in sub-Saharan Africa.

Response 2: Thank you again for your interesting comment. We have generated a pooled weight and used it throughout our analysis and we have indicated this issue as per your recommendation in the revised manuscript.

Other points for consideration:

Comment 3: Need to correct errors in some factual statements – e,g. first paragraph of introduction '…the services received after first trimester are considered late' . Do you mean to say initiation/booking of ANC after first semester is considered late?

Response 3: Thank you and sorry for the grammatical error. We have replaced it with the suggested phrase.

Comment 4: It is suggested that '…evidence on the level of late antenatal care booking and its determinants in SSA following the implementation of the 2016 new antenatal care model is limited', but it is unclear how the new ANC model deviates from previous guidelines in relation to initiation/timing of ANC.

Response 4: Thank you for your important comment. As stated in paragraph 5 of an "Introduction" section, the new recommendation deviates from the former one by the minimum number of ANC visits that a pregnant woman should attend during pregnancy. For instance, while the former model recommends a minimum of four visits, the new guideline suggests a minimum of eight visits.

Comment 5: Table 6 presents 'multilevel fixed-effects' analysis –significance for some of the factors where ORs suggest significance are not marked so, e.g rural residence and decision on healthcare. Are these omissions or intended? Clarification needed if the latter.
Response 5: Thank you very much for your important comment. It is an editorial error and we have marked them in the revision.

Comment 6: Reference to table 3 in results section, yet there is no Table 3 among the list of tables provided at the end of the paper. Is this the same as table 6?
Response 6: Thank you for your concern. Yes, it is the same as table 6 and we have corrected this issue in the revised manuscript.

Comment 7: Aspects of discussion could be better thought through. For instance, the lower prevalence of late ANC compared to other studies in this paper has been attributed to differences in sample size and scope of study, the current study having a much larger sample covering a wider geographic area. It is important to note that a larger sample size in itself is unlikely to lead to lower prevalence. Potential explanations may include use of different definition for late ANC – other studies (e.g. [14] defined late ANC as 2nd or 3rd trimester (after 1st trimester – i.e. 3 months) rather than after 4 months used in the study.
Response 7: Thank you for your important recommendation. We have considered the suggested explanation/justification for the observed discrepancy in the revision.

Comment 8: Reporting of results could be better thought through. Eg it is stated that the analysis 'revealed that health insurance coverage [AOR (95% CI) = 1.84 (1.68, 2.01)] and higher birth order 184 [AOR (95% CI) = 1.40 (1.34, 1.47)] were associated with higher odds of late antenatal care booking' without clarifying what is being compared or what the Odds ratios refer to.
Response 8: Thank you very much. We have revised this concern and provided the reference category (comparison group) for the stated factors.
