# Supplementary material for: Late initiation of antenatal care visit amid implementation of new antenatal care model in Sub-Saharan African countries: A multilevel analysis of multination population survey data
Source: PLoS One. 2025 Jan 31;20(1):e0316671. doi: 10.1371/journal.pone.0316671 (PMC11785278; doi:10.1371/journal.pone.0316671)
Supplement: Attachment — Submitted filename: reviewers comments.pdf [file pone.0316671.s004.pdf]

Dear Editors and Authors,

Thank you for giving me the opportunity to review the manuscript, titled “Late initiation of antenatal care visit amid implementation of new antenatal care model in Sub-Saharan African countries: a multilevel analysis of multination population survey data”.

The authors of the study chose a very important topic related to maternal health in Africa. This study aimed to determine the pooled prevalence of late ANC visit and its determinants among women in Sub-Saharan Africa (SSA) using DHS conducted after the implementation of new guidelines in 2016.

The authors used from 16 SSA between 2018 and 2022, with a weighted sample of 101,983 women who had ANC visits during their pregnancy. The authors applied logistic regression models for multivariate results and found the pooled prevalence of late ANC visits to be 32.0% (min 13.3% in Liberia to max 50.0% in Nigeria). The determinants of late ANC visit were women’s age and education, partner’s education, health insurance coverage, birth order, household wealth, age at marriage, decision on health care, residence, and community-level women’s illiteracy.

My assessment of the manuscript is that it is well written and is well organized. I also see that this is the second reviewer of the manuscript which has improved after the authors addressed the previous reviewers’ comments. The findings from this study have the potential to impact health interventions and policy to improve initiation of timely ANC visit and maternal health services in developing countries.

Apart from few grammatical errors, which I have mentioned below and the authors need to address them, the manuscript can be considered for publication.

The authors may opt to use ANC visit instead of ANC booking, as currently they have used the two terms interchangeably, which can be confusing. Further, the women reported their ANC visit(s) rather than their booking of ANC visit(s).

The word “bias” is usually used as uncountable; therefore, in line 300 “may be recall bias” instead of “may be a recall bias” should be used.

In line 295 “This data was collected” to be changed to “The data were collected”.

The above are few errors I have noticed; the authors should check the English language and grammar for their work in this study.
