# Supplementary material for: Late initiation of antenatal care visit amid implementation of new antenatal care model in Sub-Saharan African countries: A multilevel analysis of multination population survey data
Source: PLoS One. 2025 Jan 31;20(1):e0316671. doi: 10.1371/journal.pone.0316671 (PMC11785278; doi:10.1371/journal.pone.0316671)
Supplement: Attachment — Submitted filename: Response to Reviewers.pdf [file pone.0316671.s005.pdf]

Dear Academic Editor,

Thank you again for the opportunity to revise our manuscript "***Late initiation of antenatal care visit amid implementation of new antenatal care model in Sub-Saharan African countries: a multilevel analysis of multination population survey data (ID: PONE-D-24-21779R2)***". We have addressed the concerns raised by the reviewers using a point-by-point response as stated below. The amendments made to the manuscript have been presented using track change in the attachment titled "Revised Manuscript with Track Changes".

Response to Editor's comments

Journal Requirements:

Comment 1: Please review your reference list to ensure that it is complete and correct. If you have cited papers that have been retracted, please include the rationale for doing so in the manuscript text, or remove these references and replace them with relevant current references. Any changes to the reference list should be mentioned in the rebuttal letter that accompanies your revised manuscript. If you need to cite a retracted article, indicate the article's retracted status in the References list and also include a citation and full reference for the retraction notice.

Response 1: Thank you. We have checked the reference list, confirmed that all references are complete and correct, and ensured that we have not used a retracted paper in the citation.

Additional Editor Comments:

Comment 1: Definition of the outcome – WHO recommends 1st ANC visit within 3 months (1st trimester). In the manuscript, the authors defined late ANC after 4 months of gestation. According to the literature, ANC visit within 4 months is also considered late ANC. Additionally, authors compared their results with papers mostly discussing late ANC after 3 months. I ask the authors to revisit WHO recommendations for ANC timing and revise their results accordingly. I also suggest the authors can consult and add the following citations which detail late ANC in another low-income country

- https://journals.plos.org/plosone/article?id=10.1371/journal.pone.0309300
- https://www.dovepress.com/sociodemographic-predictors-of-initiating-antenatal-care-visits-by-pre-peer-reviewed-fulltext-article-IJWH

Response 1: Thank you very much for your important and interesting recommendation. We have changed the definition for ANC timing, did a reanalysis, and revised the result section as per your suggestion. In addition, we have provided the suggested citations for the definition of late ANC visit.

Comment 2: In the Abstract add the abbreviation SSA from the first paragraph (line 25).
Response 2: Thank you. We have added the abbreviation for sub-Saharan Africa in line 25 of the abstract section.

Comment 3: In Abstract add the full form (DHS) from the first paragraph (line 27).
Response 3: Thank you have added the extended form of DHS in line 27 of the revised manuscript.

Comment 4: Methods described aren't the ones opted by the author. They were done part of the National Surveys. These methods should be supported be relevant citations (lines 94-100).
Response 4: Thank you very much. We have provided citation for DHS methodology as suggested in the revised manuscript.

Comment 5: Dependent variable: Add citation considering my earlier comment.
Response 5: Thank you. We have added the citations suggested in comment 1 for the dependent variable.

Response to Reviewer #4 Comments

Comment 1: My assessment of the manuscript is that it is well-written and is well organized. I also see that this is the second reviewer of the manuscript which has improved after the authors addressed the previous reviewers' comments. The findings from this study have the potential to impact health interventions and policy to improve the initiation of timely ANC visits and maternal health services in developing countries. Apart from a few grammatical errors, which I have mentioned below and the authors need to address them, the manuscript can be considered for publication.

Response 1: Thank you for your appreciation and important concern. We have thoroughly checked and fixed the manuscript for grammatical and typos errors.

Comment 2: The authors may opt to use ANC visit instead of ANC booking, as currently, they have used the two terms interchangeably, which can be confusing. Further, the women reported their ANC visit(s) rather than their booking of ANC visit(s).

Response 2: Thank you for your suggestion. We have replaced "ANC booking" with "ANC visit" throughout the revised manuscript.

Comment 3: The word "bias" is usually used as uncountable; therefore, in line 300 "may be recall bias" instead of "may be a recall bias" should be used.

Response 3: Thank you for your concern. We have changed "may be a recall bias" to "may be recall bias" in the revised manuscript.

Comment 4: In line 295 "This data was collected" to be changed to "The data were collected".

Response: Thank you very much. We have replaced "This data was collected" with "The data were collected" as suggested in the revised manuscript.

Comment 5: The above are a few errors I have noticed; the authors should check the English language and grammar for their work in this study.

Response 5: Thank you for your recommendation. We have checked and fixed grammatical and editorial errors throughout the revised manuscript.
