# Supplementary material for: Late initiation of antenatal care visit amid implementation of new antenatal care model in Sub-Saharan African countries: A multilevel analysis of multination population survey data
Source: PLoS One. 2025 Jan 31;20(1):e0316671. doi: 10.1371/journal.pone.0316671 (PMC11785278; doi:10.1371/journal.pone.0316671)
Supplement: Attachment — Submitted filename: Response to Reviewers.docx [file pone.0316671.s002.docx]

**Dear Academic Editor,**

Thank you for the opportunity to revise our manuscript “***Late initiation of antenatal care visit amid implementation of new antenatal care model in Sub-Saharan African countries: a multilevel analysis of multination population survey data (ID: PONE-D-24-21779)***”. We have addressed the concerns raised by the reviewers using a point-by-point response as stated below. The amendments made to the manuscript have been presented using track change in the attachment titled “Revised Manuscript with Track Changes”.

**Journal Requirements**

**Comment 1:** When submitting your revision, we need you to address these additional requirements. Please ensure that your manuscript meets PLOS ONE's style requirements, including those for file naming. The PLOS ONE style templates can be found at <https://journals.plos.org/plosone/s/file?id=wjVg/PLOSOne_formatting_sample_main_body.pdf> and <https://journals.plos.org/plosone/s/file?id=ba62/PLOSOne_formatting_sample_title_authors_affiliations.pdf>.

**Response 1:** Thank you. We have amended the manuscript as per the PLOS ONE style requirements.

**Comment 2:** Thank you for uploading your study's underlying data set. Unfortunately, the repository you have noted in your Data Availability statement does not qualify as an acceptable data repository according to PLOS's standards. At this time, please upload the minimal data set necessary to replicate your study's findings to a stable, public repository (such as Figshare or Dryad) and provide us with the relevant URLs, DOIs, or accession numbers that may be used to access these data. For a list of recommended repositories and additional information on PLOS standards for data deposition, please see <https://journals.plos.org/plosone/s/recommended-repositories>.

**Response 2:** Thank you very much for your recommendation. We have provided the relevant URL for the data used in the study and uploaded the minimal dataset used for the analysis.

**Comment 3:** Please review your reference list to ensure that it is complete and correct. If you have cited papers that have been retracted, please include the rationale for doing so in the manuscript text, or remove these references and replace them with relevant current references. Any changes to the reference list should be mentioned in the rebuttal letter that accompanies your revised manuscript. If you need to cite a retracted article, indicate the article’s retracted status in the References list and also include a citation and full reference for the retraction notice.

**Response 3:** Thank you very much. We have checked the reference list and ensured that the list does not contain a retracted paper.

**Responses to Reviewer #1 Comments**

**Comment 1:** Line 78-80: “Furthermore, comprehensive health system and community-level interventions have been implemented to bolster the quality and accessibility of ANC services and community awareness about the importance of timely ANC initiation, respectively” The authors should provide citations and references to back this statement.

**Response 1:** Thank you very much for your important recommendation. We have provided citations and references for the stated statement as suggested.

**Comment 2:** Results: I will suggest the author report the proportion or the percentages only without quoting the number of participants.

**Response 2:** Thank you for your suggestion. We have incorporated this suggestion into the descriptive results of the revised manuscript.

**Comment 3:** Where are the tables the authors are referring to in their results? Tables should be placed directly after the next reporting result from the table. Table 1 should be quoted in the text where the results are reported.

**Response 3:** Thank you for your concern. We agree that tables should be placed right after their respective descriptive results, however, as per the journal requirements, tables are placed at the end of the main body of the manuscript just following the reference list. Thus, details of the tables quoted in the main body are available at the end of the manuscript.

**Comment 4:** Discussion-though the authors make a comparison between their findings and those of previous studies, it will be important they provide the public health implication of their findings.

**Response 4:** Thank you very much. We have provided the public health implication of the study findings under the conclusion section of the revised manuscript.

**Comment 5:** The tables on multilevel analysis results provided by the authors are difficult to understand, I will suggest the authors make their tables as concise as possible showing all the three models of the multilevel regression carried out.

**Response 5:** Thank you very much for your important recommendation. We have presented the results of multilevel regression analysis for the three models as suggested in the revised manuscript.

**Responses to Reviewer #2 Comments**

This is an insightful study that addresses the hurdle of maternal health, which is late initiation of ANC by using the latest model.

**Comment:** The manuscript is readable, however, the conclusion is too general and I would suggest authors get it edited.

**Response:** Thank you for your interesting suggestion. We have revised the conclusion section as suggested.
